# Impact of Conventional and Advanced Techniques on Stability of Natural Food Colourants

**DOI:** 10.3390/foods14183187

**Published:** 2025-09-12

**Authors:** Divya, Shruti Joshi, Jayadeep Appukuttan, Jayani Chandrapala, Mahsa Majzoobi

**Affiliations:** 1 Department of Food Technology, School of Science, RMIT University, Melbourne, VIC 3000, Australia; s4080298@student.rmit.edu.au (D.); jayani.chandrapala@rmit.edu.au (J.C.); 2 Academy of Scientific and Innovative Research (AcSIR), Ghaziabad 201001, India; shruti@cftri.res.in (S.J.); jayadeep@cftri.res.in (J.A.); 3 CSIR—Central Food Technological Research Institute, Mysuru 570001, India

**Keywords:** food colourants, stability, green techniques, synthetic colours, natural colours

## Abstract

Natural food colourants are gaining momentum in the food industry due to their clean-label appeal, safety, and potential health benefits. However, their practical application is often constrained by instability under environmental stressors such as pH fluctuations, heat, light, and oxygen. In response, both traditional and innovative strategies have emerged to improve pigment stability, with some studies reporting up to 50–80% retention of colour intensity under optimised conditions. Most existing research focuses on extraction, with limited emphasis on post-processing stability. This article reviews a wide range of food processing strategies aimed at enhancing the stability of natural pigments. It covers conventional and emerging approaches, including natural chemical stabilisers such as co-pigments, antioxidants, and metal ion chelators, physicochemical methods such as micro- and nanoencapsulation using biopolymers, and physical interventions involving drying technologies, particle size modification, and protective packaging. Modern technologies such as high-pressure processing, pulsed electric fields, ultrasound, and cold plasma are discussed as promising non-thermal alternatives, demonstrating 20–70% improvement in pigment retention compared to untreated controls. By integrating these diverse approaches, this article highlights current advancements, identifies knowledge gaps, and discusses future directions to support the development of stable, sustainable, and functional natural colourant systems for next-generation food products. Collectively, these approaches demonstrate significant potential to improve the performance and resilience of natural pigments in complex food systems.

## 1. Introduction

Colour, along with size and shape, is a key visual attribute that strongly influences consumer perception, particularly in assessing product quality and freshness at the point of purchase. With growing demand for food, the processing industry is increasingly prioritising products that are not only visually appealing but also offer health benefits.

**Figure 1:**
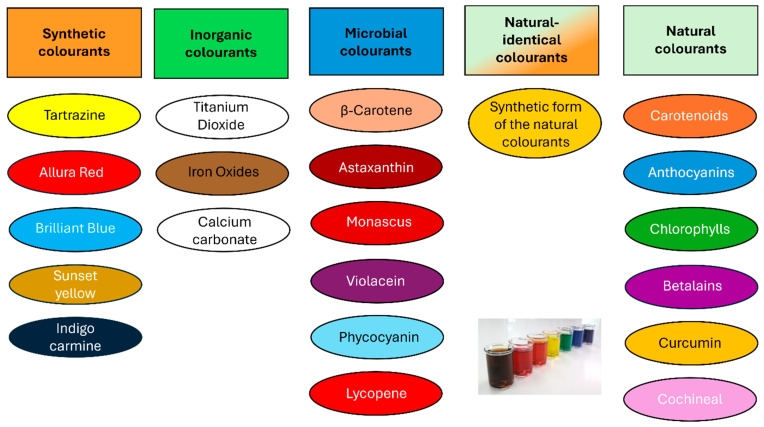
Different categories of food colourant.

Food colourants can be categorised into five main types as shown in Figure 1, natural, inorganic (mineral-based), synthetic, nature-identical, and microbial colourants [1,2]. Synthetic colourants, including tartrazine and carmine, are chemically produced and widely used for their strong colouring power and stability. Inorganic colourants, such as titanium dioxide and iron oxides, are mineral-based pigments valued for their high opacity and durability. Nature-identical colourants are chemically synthesised but structurally identical to naturally occurring compounds, like synthetic lycopene. Natural colourants are derived from plant, animal, or mineral sources (e.g., beetroot red, turmeric, chlorophyll), offering a clean-label appeal but often with lower stability. Microbial colourants are produced through microbial fermentation (e.g., Monascus pigments), offering a sustainable and potentially safer alternative.

While synthetic colourants remain common in industrial applications, growing consumer awareness and concern over artificial additives have shifted market preference toward safer, plant-based ingredients. As a result, the demand for natural, safe, and clean-label colourants is rapidly increasing, prompting food manufacturers to explore natural alternatives that align with health-conscious and chemical-free labelling trends [3,4].

Despite the rising demand for clean-label products, natural food colourants continue to face significant challenges that limit their widespread adoption. Compared to synthetic food colourants, they are inherently less stable and highly sensitive to environmental factors such as light, heat, pH fluctuations, and oxygen, often resulting in colour loss or inconsistency during processing and storage. Their extraction and purification are also costly and yield relatively low quantities, which drives up production expenses. In addition, natural food colourants can impart undesirable flavours or odours, offer a restricted colour range, and exhibit variability linked to their botanical source and harvest conditions. These drawbacks collectively hinder manufacturers in achieving the consistency, scalability, and shelf-life required for commercial food products [2,3,4,5,6,7].

To address the limitations of natural food colourants, both conventional and advanced processing technologies have been applied to protect pigments from degradation caused by heat, light, pH fluctuations, shear, and pressure [2,6]. These approaches improve colour stability, extend shelf life, and broaden the application of natural pigments across beverages, dairy products, baked goods, confectionery, processed meats, sauces, nutraceuticals, and dietary supplements. However, research remains fragmented, particularly regarding green and non-thermal strategies, and most studies focus primarily on extraction efficiency rather than preserving colour integrity, structural stability, and bioactivity. Traditional methods such as co-pigmentation, antioxidant incorporation, and encapsulation have not been systematically compared with emerging non-thermal technologies, leaving gaps in understanding their relative effectiveness and potential synergies [5,7].

This review provides a novel, integrated perspective by shifting the emphasis from extraction to stability and functionality. It critically evaluates a wide spectrum of stabilisation strategies, explores their mechanisms, and offers comparative insights across conventional and advanced methods. Importantly, it also addresses industrial scalability, regulatory frameworks, consumer perspectives, and environmental sustainability, providing guidance for practical implementation and commercial adoption. By synthesising mechanistic, technological, and application-focused knowledge, the review offers a comprehensive roadmap for the development of next-generation, clean-label, and nutritionally enhanced food products, positioning it at the forefront of natural colourant research.

### 1.1. Literature Search and Data Collection

To compile this review article, a structured literature search was conducted across major scientific databases, including Scopus, Web of Science, PubMed, and Google Scholar. Keywords such as “natural food colourants,” “pigment stability,” “microencapsulation,” “non-thermal processing,” “high pressure homogeniser,” “pulse electric field,” “ultrasound,” and “cold plasma” were combined using Boolean operators. Only peer-reviewed articles published in English from 2000 onwards were considered. Studies were selected based on relevance to pigment stability, industrial applications, and non-thermal or encapsulation techniques. Data from the selected articles were extracted and critically synthesised, focusing on mechanisms of stabilisation, environmental factors, and technological approaches. This methodology ensured a systematic, transparent, and reproducible compilation of current knowledge.

#### 1.1.1. Classification and Characteristics of Food Colourants

Understanding the physicochemical properties of food colourants are essential for ensuring their stability, functionality, and compatibility within complex food systems. Properties such as solubility, thermal stability, pH sensitivity, and light resistance directly influence colour retention, shelf life, and overall product quality. By characterising these attributes, food technologists can predict the behaviour of pigments during processing and storage, optimise their application in diverse food matrices, and select appropriate stabilisation methods. Moreover, a thorough understanding of physicochemical characteristics supports the development of clean-label, natural colourants that meet consumer expectations for safety, visual appeal, and sustainability [8,9,10,11]. Table 1 summarises the main features of natural, nature-identical, and synthetic food colourants, alongside inorganic and microbial sources.

#### 1.1.2. Food Colourants: Origins, Key Properties and Safety

Synthetic colourants are artificially produced substances added to food to enhance or restore colour lost during processing. Unlike natural colourants derived from plants or animals, synthetic colourants are created through chemical synthesis or modification of precursor compounds. They are classified into various categories, including azo dyes (e.g., Tartrazine, Sunset Yellow FCF) and triarylmethane dyes (e.g., Brilliant Blue). These colourants are more stable than natural alternatives when exposed to heat, light, or other chemicals, and they typically do not affect taste while delivering strong, vibrant hues [8]. Synthetic food colourants, while approved for use in many countries, can sometimes cause adverse effects in certain individuals, especially when consumed in large amounts or over long periods. Studies have linked dyes such as Tartrazine and Brilliant Blue FCF to hyperactivity and learning impairments in children. Some individuals may also experience allergic responses to synthetic colourants, necessitating careful monitoring and labelling to protect sensitive consumers [9].

In addition to synthetic colourants, inorganic (mineral-based) food colourants such as titanium dioxide and iron oxides are widely used for opacity and brightness, particularly in confectionery, coatings, and powdered mixes. These are generally stable and cost-effective but have come under scrutiny for safety concerns, particularly in nanoparticle form. Additionally, microbial colourants, obtained through microbial fermentation (e.g., Monascus, algae-based pigments), are emerging as promising natural alternatives due to their renewable nature, scalability, and reduced environmental impact. Their use is expanding in clean-label and functional food applications [2,9].

Nature-identical food colourants are synthetically produced compounds that are chemically identical to those found in nature. They offer greater stability, cost-efficiency, and consistent colouring including improved heat, light, and pH stability, while maintaining a more natural image. Examples include beta-carotene (orange), riboflavin (yellow), and lycopene (red), which are commonly used in drinks, dairy, and confectionery products. However, because they are synthetised, their synthetic origin may still be a concern for clean-label consumers, and regulatory recognition of them as “natural” varies by region [1,2,3].

Natural food colourants or pigments are compounds that naturally give colour to plant and animal tissues. These include substances present in leaves, fruits, vegetables, flowers, as well as in the skin, eyes, and internal tissues of animals, and in microbes such as bacteria and fungi [2,3]. Animal-based colourants include astaxanthin, haemoglobin, and myoglobin extracted from crustacean shells and animal blood or muscle. While these can provide essential nutrients like bioavailable heme iron, overconsumption of highly processed meats containing such pigments has been linked to chronic disease risks (e.g., colorectal cancer). In food products, natural pigments are generally classified into four main types: anthocyanins (red, blue, purple), betalains (red), chlorophylls (green), and carotenoids (yellow, orange, red) [10,11,12]. For instance, carotenoids, commonly found in fruits and vegetables, are valued for their vibrant colour and antioxidant activity, and play an important role in improving the visual and nutritional quality of foods and beverages [11]. Common features of natural food colourants are listed in Table 2.

Safety assessments are a critical component in determining the regulatory approval of food colourants. Many natural pigments undergo rigorous evaluation to establish their safety for human consumption, including toxicological studies, allergenicity tests, and metabolic fate assessments [12]. In the United States, natural colourants must obtain Generally Recognized as Safe (GRAS) status, either through FDA review or self-affirmation based on scientific evidence [13]. Similarly, in the European Union, EFSA evaluates the safety of both natural and synthetic colourants, setting acceptable daily intake (ADI) limits to guide safe use [7,14]. India’s FSSAI also requires safety validation, particularly for newer natural extracts introduced as food colourants [15,16,17]. These evaluations ensure that both natural and synthetic pigments meet stringent safety standards before entering the consumer market, supporting confidence in their use across different food applications.

As awareness of health risks linked to synthetic food dyes increases, natural pigments are being explored as safer options. For example, betalains have shown great potential as alternatives to Allura Red AC (Red 40), a synthetic dye that contains benzidine, a chemical which associated with possible cancer risks in humans and animals [11]. Another example, the use of cochineal (E120), a red dye derived from the cochineal insect, has increased, along with the adoption of Beetroot Red (E162) and chlorophylls (E140) for red and green colouring, respectively. However, natural dyes are also subject to food safety evaluations, and some additives are substituted with safer alternatives [18,19,20].

Some natural food colourants also offer significant health benefits, enhancing their appeal in the food industry. Naturally occurring pigments such as carotenoids and flavonoids possess notable antioxidant activity, aiding in the neutralisation of free radicals and the mitigation of oxidative damage within the body [19]. They contribute to eye health and have antibacterial properties, further enhancing their health-promoting potential. These compounds also exhibit anti-inflammatory effects, which may contribute to lowering inflammation and the risk of chronic illnesses, while also providing neuroprotective benefits that could help prevent neurodegenerative disorders. Certain pigments, such as delphinidin, have shown significant anticancer activity by inhibiting the growth of cancer cell lines. Additionally, they are used in innovative applications such as intelligent food packaging, which can monitor food freshness and quality [4]. Despite this, the widespread adoption of natural pigments faces obstacles such as molecular instability, higher production costs compared to synthetic alternatives, the need for larger quantities to achieve similar colour intensity, and a more restricted range of colours [10].

#### 1.1.3. Regulatory Framework for Food Additives

EU Regulation (EC) No 1333/2008 governs the use of food additives as ingredients in the production or preparation of food [7]. In Europe, all food additives are given a specific code that begins with the letter “E” followed by three or four digits, referred to as the E-number. This coding system makes it easier for the consumer to understand the label of foodstuffs across different European countries. The same code number is also used by the Codex Alimentarius [20]. According to Annex I of Regulation (EC) No 1333/2008, “Colours are substances which add or restore colour in a food, and include natural constituents of foods and natural sources which are normally not consumed as foods as such and not normally used as characteristic ingredients of food’’ [7,20].

The minimum required levels of colour compounds differ widely among colourants. For some, such as anthocyanins, no specific minimum content is established. In contrast, others are strictly defined: betanine must contain at least 0.4%, plant-derived carotenes at least 5%, chlorophyll a minimum of 10%, while curcumin can reach concentrations as high as 95% [7]. In India, the Food Safety and Standards Act (2006) prescribes food regulations that are implemented by the Food Safety and Standards Authority of India (FSSAI), which is authorised and functions under the Ministry of Health and Family Welfare of India. FSSAI has published the list of natural and synthetic colourants with permitted limits in 2 regulations [15,16,17]. As per FSSAI regulations, the final concentration of synthetic food colourants should not exceed 100 ppm in foods and beverages [13,14,15].

In the United States, food additives and colourants are regulated under the Federal Food, Drug, and Cosmetic Act, and their use is monitored by the U.S. Food and Drug Administration (FDA) [13]. The FDA classifies colour additives into two main groups: those subject to certification (such as FD&C synthetic dyes) and those exempt from certification (typically derived from natural sources). Each approved colourant must meet strict usage limits, including maximum allowable concentrations depending on the food type. For example, FD&C Red No. 40 (Allura Red) has specific limits, such as 200 mg/kg in certain beverages [13].

In Australia and New Zealand, food colourants are governed by Food Standards Australia New Zealand (FSANZ) under the Australia New Zealand Food Standards Code. This regulation outlines permitted colourants in Standard 1.3.1 [21], listing both synthetic and natural colour additives, with assigned INS numbers. FSANZ sets maximum use levels based on risk assessments and intended use within food categories [21].

Globally, other countries follow similar frameworks. The European Union, for instance, operates under Regulation (EC) No 1333/2008, enforced by the European Food Safety Authority (EFSA), which defines permitted food colourants and their acceptable daily intakes [14]. Canada regulates colour additives through Health Canada’s Food Directorate [22], while Japan follows its Food Sanitation Law, managed by the Ministry of Health, Labour and Welfare (MHLW) [23]. Most of these authorities refer to international safety assessments and guidelines from the Codex Alimentarius Commission, which provides harmonised food additive standards [18,19,20].

Table 3 presents a representative selection of commonly used natural and synthetic food colourants, highlighting their maximum allowable concentrations, regulatory authorities, and corresponding E-, FD&C, or INS codes. It does not include all colourants globally, as regulations and permitted limits can vary across countries, and some minor or region-specific additives may not be widely used. Additionally, maximum allowable concentrations often differ depending on the specific food category, which is beyond the scope of this summary. The table is intended to provide a clear comparative overview rather than an exhaustive listing of all authorised colourants worldwide.

#### 1.1.4. Consumer Perception of Food Colourants

Consumer perception of “natural” food colourants varies significantly across regions due to differences in regulatory definitions and labelling practices. In the European Union, products labelled as “natural” must comply with strict criteria outlined in Regulation (EC) No 1333/2008, which recognises only substances derived from natural sources and minimally processed [7,14]. In contrast, the United States allows broader interpretation under FDA guidelines, where colourants derived from natural sources can be marketed as “natural” even if subject to certain processing steps [13]. In many Asian countries, including India and Japan, consumer understanding of “natural” is influenced by both traditional usage and regulatory guidance, but awareness and trust in natural labelling are still developing [15,16,23]. These regional differences can affect consumer acceptance, purchasing behaviour, and the market potential of natural colourants in global food products. The regulatory landscape for food colourants varies across regions, with different authorities setting maximum allowable concentrations and using distinct coding systems.

#### 1.1.5. Opportunities and Drawbacks of Natural Food Colourants in Food Systems

As demand for natural food colourants continues to rise, it is crucial to understand both their advantages and limitations to support their effective use within the food industry. For instance, anthocyanins are water-soluble pigments found in fruits and vegetables like berries and red cabbage, exhibiting pH-dependent colour variations. They offer various health benefits, including antioxidant, anti-inflammatory, anti-obesity, antidiabetic, and anticancer effects [4]. However, they are unstable when exposed to pH fluctuations, heat, and light [3], which can lead to undesirable colour shifts. At high concentrations, anthocyanins may also impact the flavour profile of food products [23,24].

Many natural pigments are prone to instability, yet some show performance comparable to synthetic dyes. These pigments offer additional advantages, including enhanced safety and potential health benefits. Beyond their colour and sensory appeal, several natural additives also function as antioxidants or preservatives. This allows them to play multiple functional roles in food products [12]. The main advantages and disadvantages of using natural food colourants are highlighted in Table 4.

Natural pigments often face stability challenges due to their sensitivity to factors such as pH, temperature, light, and concentration. Enhancing their durability and performance in food applications is therefore crucial. By understanding the physicochemical and environmental factors that influence pigment stability, targeted strategies can be developed to improve their resilience. This section explores various stabilisation techniques and technological approaches that help maintain colour, extend shelf life, and preserve the functional properties of natural food colourants, enabling their wider and more reliable use in the food industry [18,19].

#### 1.1.6. Relationship Between Pigment Structure and Stability

The stability of pigments is intricately linked to their molecular architecture, influencing their susceptibility to environmental factors such as pH, temperature, light, and chemical agents. It has been shown that anthocyanins, water-soluble flavonoid pigments, exhibit varying stability based on their glycosylation and acylation patterns. For instance, anthocyanins from *Clitoria ternatea* (blue pea) demonstrate enhanced stability due to specific sugar and acyl group positioning, which affects their protonation states and quinoidal base formation [4]. Carotenoids, such as β-carotene and lutein, possess conjugated double bonds that are susceptible to oxidation, leading to degradation. The introduction of hydroxyl or epoxy groups can improve their stability by reducing susceptibility to oxidative damage [19]. Betalains, nitrogen-containing pigments, are more stable under acidic conditions. Their stability is influenced by the presence of glycosylation and the nature of the nitrogenous substituents. Chlorophylls, responsible for the green colour in plants, are prone to degradation under light and heat. Their stability can be enhanced by esterification and chelation with metal ions, which protect the porphyrin ring from oxidative damage [10].

#### 1.1.7. Assessment of Natural Colourant Stability: Key Parameters and Methods

Evaluation of pigment stability is critical to determine the effectiveness of these methods. Key parameters include colour retention, pigment degradation rates, solubility, antioxidant activity, and response to environmental stressors such as temperature, pH, and light exposure. Common techniques to assess stability include spectrophotometry for colour measurement, chromatography for compound profiling, and accelerated shelf-life studies to simulate storage conditions [7,12]. Understanding these parameters allows optimisation of stabilisation strategies and ensures pigments maintain their functional and aesthetic properties in food systems. Table 5 presents advanced characterisation techniques used to evaluate the stability and efficacy of natural food colourants. These methods provide insights into pigment structure, interactions with matrices, and resistance to environmental stressors, thereby supporting informed selection of stabilisation strategies in food applications [28,29,30,31,32].

#### 1.1.8. Physicochemical Factors Affecting the Stability and Efficacy of Natural Food Pigments

The efficacy and stability of natural pigments are strongly influenced by their physicochemical environment during processing, storage, and application. pH plays a critical role, particularly for anthocyanins and betalains, where acidic conditions generally enhance stability, whereas neutral or alkaline conditions can trigger degradation and colour loss [7]. Temperature is another key determinant; elevated heat can induce pigment breakdown, isomerisation, or oxidation, while non-thermal techniques such as HPP, ultrasound, and cold plasma can help preserve colour by minimising thermal stress [29]. Light exposure, especially ultraviolet and visible spectra, can catalyse photochemical reactions in curcumin, carotenoids, and chlorophylls, leading to colour fading; incorporating UV-absorbing additives or protective packaging effectively mitigates these effects [30,31].

Oxygen availability is pivotal for oxidative degradation. Strategies such as vacuum packaging, modified atmosphere packaging, and edible coatings limit oxygen contact, preserving both pigment integrity and associated bioactive properties [28,31]. The molecular structure of pigments, including conjugation, glycosylation, acylation, and substitution patterns, determines susceptibility to chemical and enzymatic breakdown, while interactions with proteins, polysaccharides, and encapsulating matrices can enhance stability through complex formation [32,33].

Finally, water activity and moisture content influence hydrolytic reactions and pigment mobility, affecting stability in powders and liquid formulations. Encapsulation and particle size reduction improve dispersion and reduce exposure to degradative agents, optimising pigment retention and bioavailability [32,33]. Collectively, understanding these physicochemical parameters is essential for selecting suitable stabilisation strategies and designing robust food systems that maintain the functional and aesthetic properties of natural colourants.

#### 1.1.9. Stabilisation Techniques for Food Colourants

The stability of natural food colourants is a critical concern in food formulation, as they are highly susceptible to degradation from environmental factors such as pH changes, temperature fluctuations, and light exposure. Additionally, oxygen and enzymatic activity can further compromise their integrity, posing challenges for maintaining consistent colour and functionality in food products [28]. For instance, anthocyanins demonstrate pronounced pH-dependent transformations, while carotenoids are highly vulnerable to oxidation due to their unsaturated structure and hydrophobic nature [28,30]. Traditional methods for stabilising natural food colourants have largely focused on environmental control, such as adjusting pH and temperature, alongside physical protection strategies like encapsulation and the use of antioxidants or co-pigments [30]. While these approaches are well-established and effective to a certain extent, they often involve additional chemical additives and may have limited efficacy in complex food systems. In contrast, modern techniques such as US, HPP, PEF, and IR offer innovative, non-thermal alternatives that enhance pigment stability by preserving molecular structure and functional properties without excessive heat. These advanced methods align better with clean-label trends, often providing greater energy efficiency and environmental benefits. Additionally, when combined with traditional approaches, modern techniques can yield synergistic effects, resulting in improved colour retention and extended shelf life, making them promising for broader industrial application. This section provides a comprehensive analysis of stabilisation strategies, emphasising natural, safe interventions such as green chemicals, physical methods, advanced technologies, and their combinations. These approaches align closely with the clean-label movement, prioritising minimal processing and the use of food-grade, naturally derived additives to preserve pigment integrity and functional performance throughout processing and storage.

## 2. Natural Chemical Methods

Natural chemical stabilisation strategies offer multifaceted benefits by preserving colourant integrity and aligning with consumer demand for sustainable and minimally processed foods. These strategies as explained below typically involve the incorporation of co-pigments, chelators, organic acids, natural antioxidants, and enzyme inhibitors to create a protective environment that minimises pigment degradation under various stress conditions (Table 6).

### 2.1. Co-Pigmentation and Mixing

Co-pigmentation refers to the non-covalent interaction between anthocyanins and co-pigment molecules such as phenolic acids and tannins which results in enhanced colour expression and increased pigment stability. This mechanism relies primarily on π-π stacking and hydrogen bonding interactions that stabilise the flavylium ion structure of anthocyanins, rendering them less prone to nucleophilic attack and hydration-induced discolouration [34,35]. Pigments like anthocyanins form non-covalent complexes with co-pigments, stabilising their molecular structure and reducing degradation from pH, light, and oxygen [12,34]. Antioxidants neutralise free radicals, and metal chelators sequester transition metals that catalyse oxidative reactions, protecting pigments from oxidative damage [12].

Recent studies highlight the role of specific phenolic acids such as caffeic, ferulic, and p-coumaric acids in producing hyperchromic effects and bathochromic shifts, significantly improving both colour vibrancy and resistance to environmental degradation [36]. Notably, acylated anthocyanins demonstrate superior stability in fluctuating pH environments and in the presence of transition metal ions such as Fe^2+^ and Fe^3+^ due to steric hindrance provided by the acyl groups [37].

Beyond colour enhancement, co-pigmentation also imparts improved antioxidant and antibacterial properties, thereby contributing to the functional profile of food products [35]. Practical applications have emerged in smart packaging systems, where co-pigmented films incorporating tannin–anthocyanin complexes respond to environmental stimuli such as pH or volatile amines, enabling real-time monitoring of food spoilage [35].

Nevertheless, the efficacy of co-pigmentation is contingent upon multiple factors including the structural characteristics of the co-pigments, the molar ratio between pigments and co-pigments, and the surrounding pH and temperature [34]. Tailored co-pigmentation strategies must be developed for different food systems to optimise these benefits.

### 2.2. Metal Ion Complexation

Metal ion complexation represents another natural strategy to enhance the structural stability of pigment molecules, particularly anthocyanins and flavonoids. The formation of stable metal pigment complexes with ions such as Al^3+^, Fe^3+^, and Mg^2+^ increases molecular rigidity, thereby improving pigment resistance to pH shifts, oxidation, and thermal degradation [38]. These complexes often exhibit a characteristic bluish-purple hue and offer prolonged shelf-life benefits under variable storage conditions. However, the stabilising effect is highly dependent on the nature and concentration of the metal ions employed. While metals like Al^3+^ may enhance stability, others like Cu^2+^ or Fe^3+^ can inadvertently catalyse oxidative reactions if not carefully balanced, underscoring the importance of precision in formulation [30].

Anthocyanins are unstable at physiological pH, but the thermal stability of cyanidin-3-glucoside (C3G) can be enhanced by Fe^3+^, though this causes aggregation. Adding anionic polysaccharides like alginate prevents aggregation and further improves stability. Similar effects were seen with delphinidin-3-glucoside but not pelargonidin-3-glucoside. These findings suggest that alginate forms a stable complex with C3G through Fe^3+^, enhancing its thermal resistance [39].

### 2.3. Protein–Polyphenol Interactions

Interactions between dietary proteins and polyphenolic colourants are gaining attention as effective stabilisation mechanisms. These interactions, facilitated primarily through hydrogen bonds and hydrophobic associations, lead to the formation of protein–polyphenol complexes that shield pigment molecules from degradation [40,41]. Dairy proteins (e.g., casein, whey) and plant-based proteins (e.g., soy, pea) have demonstrated potential in stabilising anthocyanins and other polyphenols within beverage and dairy matrices. These complexes not only protect against thermal and oxidative stress but may also modulate pigment bioavailability during gastrointestinal digestion, offering dual benefits of protection and controlled release [41].

### 2.4. pH Adjustment Using Natural Acids

Given the pH sensitivity of pigments such as anthocyanins and betalains, maintaining an acidic environment is crucial for colour stability. Natural acids including citric, malic, ascorbic, and acetic acid are widely employed in food systems to maintain a low pH, which favours the stable flavylium ion form of anthocyanins [41]. Discolouration in blanched broccoli is significantly accelerated under acidic conditions, with more hydrophobic acids causing faster pigment degradation. Additionally, at neutral to alkaline pH, chlorophyll degradation continues through a pH-independence mechanism involving magnesium ion loss, highlighting the complex role of both acid type and pH gradients in colour stability. Alkalizing agents in blanch and brine solutions, such as sodium bicarbonate, hexametaphosphate, disodium glutamate, sodium hydroxide, zinc and magnesium hydroxide, have been used to raise the pH of green vegetables and, therefore, retain chlorophyll after processing [42].

In contrast to chlorophyll, anthocyanins tend to lose colour upon heating due to a shift in equilibrium toward their colourless forms, including the carbinol base and chalcone structures. Their vivid colouration is most pronounced in strongly acidic environments, with isolated anthocyanins showing minimal colour above pH 3.5. However, in natural systems, copigmentation with other plant-derived compounds often colourless themselves enhances visible colour. Typically, at pH levels around 3 or lower, the red, orange, or purple flavylium cation is predominant. As pH increases, this cation undergoes hydration and proton transfer reactions: the former leads to colourless carbinol pseudobases and eventually yellow chalcones, while the latter results in violet quinonoidal forms. At pH 6–7, further deprotonation produces bluish quinonoid anions. Therefore, within the pH range found in most fruits and vegetables, anthocyanins exist as a dynamic equilibrium of multiple structural forms, each contributing differently to the overall colour [42]. These acids often exhibit synergistic effects, acting concurrently as antioxidants or metal chelators to further fortify pigment stability. This strategy is particularly beneficial for juice products, jams, and fermented foods, where maintaining a vibrant red or purple hue is integral to product quality.

### 2.5. Metal Chelation by Natural Agents

Trace metals such as iron and copper, even in minute concentrations, can catalyse the oxidative degradation of colourants. Natural chelating agents including phytic acid, ascorbic acid, and tannic acid are effective in sequestering these pro-oxidant metal ions, thereby preventing discolouration and preserving overall product quality [43]. Chelation is especially advantageous for pigments like curcumin and anthocyanins, which are particularly sensitive to metal-induced degradation. These chelators not only support pigment preservation but also align with clean-label and functional ingredient trends, offering antioxidant and health-promoting benefits in addition to stabilisation [39].

### 2.6. Enzyme Inhibition

Enzymatic browning, primarily catalysed by polyphenol oxidase (PPO) and peroxidase (POD), poses a major threat to the stability of natural colourants in fruits and vegetables. These enzymes oxidise phenolic compounds, leading to undesirable browning, pigment loss, and nutritional degradation. Natural inhibitors such as ascorbic acid, citric acid, and phenolic-rich plant extracts have demonstrated efficacy in curbing PPO and POD activity [44,45].

Natural and chemical strategies are commonly employed to reduce enzymatic browning in foods. Natural inhibitors like onion, pineapple, lemon juice, and honey have shown effectiveness in slowing down browning by suppressing polyphenol oxidase (PPO) activity. For instance, onion extract directly inhibits PPO, pineapple juice reduces browning in fruits like apples and bananas, and lemon juice rich in ascorbic and citric acids helps brighten baked products while limiting pigment degradation. Honey also delays browning in cut fruits. Additionally, genetic approaches, such as in Arctic apples, involve silencing PPO expression to prevent browning and enhance sensory quality. Chemical methods, including the use of inorganic salts, edible coatings, proteases, and zinc chloride, also serve as effective anti-browning agents [25,44].

Polyphenol oxidase (PPO) activity depends on copper as a cofactor, and its inhibition can be achieved through copper-chelating agents. Compounds such as citric acid, polyphosphates, sorbic acid, polycarboxylic acids, porphyrins, EDTA, and hinokitiol are commonly used in the food industry to inhibit PPO activity and prevent browning. In particular, hinokitiol is applied in food packaging coatings for its chelating and preservative properties, enhancing both colour retention and shelf-life stability [44]. Table 6 shows additional studies on these chemical methods.

**Table 6:** Natural chemical stabilisation techniques for food colourants.

Technique	Primary Pigments Targeted	Mechanism	Condition Used	References
Co-pigmentation	Anthocyanins	Non-covalent molecular interactions between anthocyanins and co-pigments (e.g., flavonoids, phenolic acids) that enhance colour intensity and stability.	Chokeberry anthocyanins with ferulic acid at a 1:172 molar ratio showed ~200% hyperchromic shift; mulberry anthocyanins with ferulic acid gave ~52.94% colour enhancement; catechins at 0.01 mL showed optimal stabilisation	[34,46,47]
Metal Ion Complexation	Anthocyanins, Chlorophylls and other metal-sensitive pigments	Coordination between pigments and metal ions (e.g., Al^3+^, Fe^2+^) that modifies pigment structure and improves stability against pH, light, and heat.	Acylated anthocyanins from Exberry^®^ exhibited higher stability at pH 3 and 6 when complexed with ferrous/ferric ions. Predictive model based on SERS developed for stability indexing.	[37]
Protein-Polyphenol Interactions	Anthocyanins, Polyphenols	Formation of stable complexes between proteins and polyphenolic pigments, enhancing thermal and oxidative stability.	Commonly used with dairy and plant proteins to bind anthocyanins in beverage matrices.	[40,41]
pH Adjustment Using Natural Acids	Anthocyanins, Betalains and other pH sensitive pigments	Maintains pigments in their most stable ionic form (e.g., flavylium cation for anthocyanins) by acidifying the medium using natural acids like citric or malic acid.	Black rice anthocyanins stabilised at pH 3.8 using catechin addition; low acidity enhances pigment preservation.	[47]
Metal Chelation by Natural Agents	Anthocyanins, Chlorophylls	Natural chelating agents (e.g., citric acid, phytic acid) bind free metal ions to prevent catalytic pigment degradation.	Gum arabic and iron-alginate demonstrated enhanced thermal and oxidative protection of anthocyanins from Ipomoea batatas.	[48]
Enzyme Inhibition	Anthocyanins, Flavonoids	Natural inhibitors (e.g., ascorbic acid, L-cysteine) reduce enzymatic browning and pigment degradation by inhibiting polyphenol oxidase.	Chokeberry anthocyanins retained stability post enzymatic treatment, indicating limited enzymatic degradation.	[46]

## 3. Physicochemical Methods

Physicochemical methods represent a convergence of physical and chemical approaches to improve stability, bioavailability, and functional performance of natural food colourants. These techniques are especially important for plant-based pigments, which are highly sensitive to oxidation, temperature fluctuations, pH changes, and light exposure. Commonly employed strategies include encapsulation at micro- and nano-scales and hydrocolloid complexation [49,50]. Emerging systems, such as Pickering emulsions, provide additional benefits by protecting pigments while enabling controlled release, targeted delivery, and enhanced sensory and therapeutic properties.

Encapsulation technologies have received increasing attention due to their capacity to preserve the chemical composition and pharmacological activity of bioactive compounds while mitigating issues such as unpleasant odour or flavour and instability in harsh environments [49]. Nanoencapsulation, in particular, offers advantages over conventional encapsulation methods due to its higher surface area, increased permeability, and improved delivery efficiency [50].

### 3.1. Micro and Nanoencapsulation

Microencapsulation is a widely employed technique that encloses bioactive substances, such as natural colourants, within protective carriers at the micron scale. Techniques such as spray-drying, freeze-drying, extrusion, emulsification, and coacervation are used to produce microcapsules, thereby protecting pigments from adverse environmental conditions such as heat, oxygen, light, and pH fluctuations [50]. This process is particularly valuable for pigments like anthocyanins, carotenoids, betalains, and chlorophylls, which are highly susceptible to degradation during food processing and storage. Nanoencapsulation provides an advanced alternative to microencapsulation by forming carriers at the nanometre scale, enhancing both the stability and bioefficacy of encapsulated compounds. These carriers include polymeric nanoparticles, lipid-based systems (e.g., nanoemulsions and liposomes), solid lipid nanoparticles, and metal-based nanocarriers such as gold nanoparticles [51,52]. Due to their small size and high surface-to-volume ratio, nanocarriers offer superior protection against light, heat, oxygen, and enzymatic degradation while promoting controlled release and targeted delivery.

In both micro and nanoencapsulation, the encapsulation in biopolymers (e.g., maltodextrin, gum arabic, chitosan) physically shields pigments from environmental stressors such as oxygen, light, and heat. Nanostructured carriers enhance barrier properties due to their high surface-area-to-volume ratios, improving solubility, dispersion, and retention of functional properties in complex food systems [4,10].

Lycopene, for example, has been successfully microencapsulated using spray-drying with modified starch as the coating agent, demonstrating improved thermal and oxidative stability. When incorporated into cake matrices, these lycopene microcapsules effectively released the pigment during baking without compromising its colour intensity or antioxidant capacity [51]. This exemplifies how microencapsulation not only stabilises pigments but also facilitates their integration into complex food systems.

Bhandari et al., 2024 [53] reported that nanoencapsulation significantly improved the thermal stability of β-carotene, α-carotene, and lutein using poly-ε-caprolactone polymers. For example, β-carotene retained 73.34% of its bioactive content post-heating when encapsulated, in contrast to only 31.64% in the unencapsulated form, clearly indicating the enhanced protection provided by the nano-matrix [53].

In another study, Zannou et al., (2023) explored various nano/microgel systems for encapsulating anthocyanins, particularly cyanidin-3-O-glucoside (C3G) Zannou, Oussou, Chabi, Awad, Aïssi, Goksen, Mortas, Oz, Proestos and Kayodé [54]. Alginate hydrogel beads coated with chitosan showed significantly higher encapsulation efficiency and stability compared to whey protein and gelatin-based systems. Furthermore, a modified chitosan derivative β-cyclodextrin-epichlorohydrin-grafted carboxymethyl chitosan demonstrated improved thermal and light resistance of C3G, providing both structural protection and enhanced antioxidant function. Beyond stability, nanoencapsulated pigments have been associated with amplified therapeutic effects, including anti-inflammatory and antioxidant properties [53]. Henao-Ardila et al., (2024) demonstrated that oil-in-water nano-emulsions of carotenoids using gelatin and whey protein effectively stabilised lipophilic pigments, offering improved solubility and bioaccessibility [55].

Importantly, nanoencapsulation also supports controlled release in food and digestion systems. For instance, anthocyanins from berries, which typically degrade under alkaline conditions, can be encapsulated in chitosan-based nanocarriers to maintain their colour and antioxidant potential during gastrointestinal transit, ultimately enhancing their effectiveness in functional beverages [56]. Despite its advantages, the commercial viability of nanoencapsulation faces challenges such as production cost, scalability, and regulatory constraints, which need to be addressed to facilitate broader adoption.

### 3.2. Hydrocolloid Complexation

Hydrocolloids high-molecular-weight biopolymers such as pectin, gum Arabic, alginate, chitosan, and agar are frequently used to form complexation systems that stabilise natural pigments through both physical entrapment and chemical interactions [38,57]. These materials can form microgels, emulsions, or hydrogel networks that trap pigments and reduce their exposure to degrading agents like oxygen, light, or extreme pH. Mechanistically, hydrocolloids stabilise pigments via non-covalent interactions including hydrogen bonding, electrostatic attractions, and hydrophobic forces. For example, anthocyanins, which exist in the flavylium cation form at low pH, can interact with negatively charged polysaccharides such as pectin or alginate, resulting in electrostatic stabilisation [38]. Conversely, chitosan a positively charged biopolymer forms electrostatic and hydrogen bonds with anionic anthocyanin forms, enhancing pigment retention and antioxidant stability [58].

FTIR analysis has revealed strong intramolecular interactions among C-phycocyanin, sucrose, and agar, correlating with improved colour retention [59]. Hydrocolloids not only stabilise colour but also modulate the textural attributes and mouthfeel of food products, enhancing sensory appeal [60]. However, their effectiveness is influenced by food matrix composition, pH, sugar content, and processing conditions.

### 3.3. Pickering Emulsions

Pickering emulsions are a novel encapsulation system where the interface between immiscible phases (e.g., oil and water) is stabilised by solid particles instead of traditional surfactants. These systems offer improved mechanical and oxidative stability due to the irreversible adsorption of solid particles at the interface, forming a robust physical barrier. De Carvalho-Guimarães et al., (2022) reviewed the use of biodegradable and biocompatible particles such as clay minerals for the stabilisation of Pickering emulsions, particularly in pharmaceutical and cosmeceutical applications [61]. These emulsions enable controlled release and enhanced skin permeability of active compounds.

In food systems, their application is growing, with interest in using natural pigments as both colourants and functional agents in delivery vehicles. Factors such as particle size, wettability, and concentration strongly affect emulsion stability, and achieving the right hydrophilic hydrophobic balance remains a formulation challenge. Pickering emulsions hold great potential for delivering food pigments. They can protect sensitive compounds, such as carotenoids and anthocyanins, during processing, while also enabling controlled pigment release. Additionally, they may be used in intelligent packaging systems, serving as colour-changing indicators [62].

Physicochemical stabilisation techniques ranging from micro- and nanoencapsulation to hydrocolloid complexation and Pickering emulsions play a vital role in enhancing the shelf life, bioactivity, and aesthetic appeal of natural colourants. These strategies not only provide protection against environmental and processing-induced stressors but also enable innovative applications such as controlled release and targeted functionality. Table 7 shows the additional studies on these physicochemical methods.

## 4. Physical Methods

Physical methods have emerged as a cornerstone for enhancing the stability of natural food colourants, particularly because many natural pigments such as anthocyanins, carotenoids, betalains, and chlorophylls are inherently unstable under typical food processing and storage conditions. Physical stabilisation strategies offer a non-chemical, process-oriented approach that aligns well with clean-label and sustainable processing goals. Techniques such as spray drying and freeze-drying lower water activity, slowing hydrolysis and enzymatic pigment degradation. Reducing particle size enhances uniform dispersion but may increase oxygen exposure if not encapsulated. Protective packaging, particularly UV-blocking films, limits light-induced oxidation and maintains colour integrity [4,10]. This section critically examines various physical techniques including particle size reduction, drying methods, edible films and coatings, protective packaging, and controlled storage conditions emphasising their mechanisms, effectiveness, and limitations in maintaining pigment integrity and visual quality across diverse food matrices.

### 4.1. Particle Size Reduction

Reduction in particle size through nanotechnology or micronisation is a promising approach to improve the dispersion, solubility, and stability of natural food colourants. Smaller particles exhibit increased surface area, enhancing their interaction with stabilising matrices while reducing exposure to degradative elements like oxygen and moisture.

In a study on carotenoid-rich carrot powders, Haas et al., (2019) [67] demonstrated that particle morphology strongly influenced both oxidative stability and colour retention. Spray-dried powders with compact, low surface-to-volume ratios retained 76–77% of carotenoids during storage, compared to 69–93% degradation in more porous freeze-dried powders, highlighting the importance of surface structure and encapsulation density in preventing pigment oxidation [67].

β-Carotene nanoemulsions developed by high-pressure homogenisation showed a reduction in droplet size from 416 nm to 97 nm under optimal processing conditions (120 MPa, three cycles), with stability maintained for up to five weeks at 25 °C [68]. Emulsions stabilised with whey protein isolate (WPI) exhibited slower β-carotene degradation than those with Tween 20, due to improved surface protection. Similarly, papaya carotenoids displayed higher bioaccessibility when droplet sizes were minimised through high-pressure homogenisation [69].

The stabilisation mechanism of particle size reduction lies in the increased surface area and reduced diffusional distance of the particles, which enhances pigment dispersion and interaction with protective matrices. Smaller particles are less prone to aggregation and allow more efficient encapsulation, thereby limiting oxygen and moisture penetration that drive oxidative degradation. Nano- and micro-scale systems also provide a physical barrier around sensitive compounds, delaying pigment breakdown and extending colour retention. Additionally, by decreasing droplet or particle size, bioaccessibility is improved through faster dissolution and enhanced interaction with digestive enzymes, contributing to both stability during storage and improved functional delivery [68,69].

### 4.2. Drying Techniques: Spray-Drying and Freeze-Drying

Spray-drying (SD) and freeze-drying (FD) are two of the most widely utilised techniques for stabilising heat-sensitive bioactives, including natural pigments. Both processes convert liquid pigment formulations into powdered form by embedding them within protective carriers such as maltodextrin, whey protein isolate, or inulin. This encapsulation reduces exposure to oxygen and light, improving pigment retention during storage and distribution [70]. The mechanism underlying SD lies in the rapid atomisation and evaporation process, where a thin film of carrier forms around pigment molecules, minimising their contact with oxygen and heat. In contrast, FD operates through ice sublimation under vacuum, which removes water at low temperatures and creates a porous matrix that better preserves structural integrity and bioactive compounds. These mechanisms explain why FD often achieves higher retention of heat-sensitive compounds, whereas SD remains more scalable and cost-effective for industrial-scale pigment encapsulation [70,71].

Ledari et al., (2024) studied microencapsulation of chlorophyll using SD and FD and found that freeze-dried microcapsules (FDM) exhibited higher ζ-potential, better thermal stability (melting point: 150.12 °C), and smaller particle sizes than spray-dried counterparts, contributing to improved pigment stability [70]. Chen et al., (2021) [71] also compared these methods for processing Liluva (pea processing water), observing that SD yielded lighter powders with higher fibre content, while FD better retained soluble carbohydrates and lysine. Despite minor nutrient losses, spray-drying was deemed more efficient for industrial applications [71].

Kucharska-Guzik et al., (2025) demonstrated that both SD and FD retained significant levels of phenolic compounds (73.2–78.5 mg/g DM) in *Cistus creticus* L. powders when using maltodextrin or inulin as carriers, with the resulting powders maintaining desirable moisture levels, antioxidant activity, and colour stability [72]. Similarly, Souza et al., (2013) reported that spray-drying Bordo grape byproducts with 30% maltodextrin at 170 °C retained about 97% of anthocyanins and improved solubility and colour stability [73], while Mahdavi et al., (2016) confirmed that a gum Arabic–maltodextrin blend effectively preserved anthocyanins in barberry extract under light exposure for 90 days [74].

### 4.3. Edible Films and Coatings

The mechanism of edible films and coatings lies in their ability to form semi-permeable matrices of biopolymers (e.g., starch, chitosan, proteins), which regulate gas and moisture transfer while also stabilising pigments by restricting exposure to oxygen, light, and pH fluctuations [30]. These biopolymer systems therefore act as multifunctional barriers against environmental stressors. For example, in red pepper powders, colour degradation (i.e., shift from red to orange) was strongly associated with initial moisture content and packaging permeability, although losses in capsaicinoids and sugars were less pronounced [75]. These findings highlight the importance of film formulation and packaging compatibility when designing systems to preserve colourant stability, particularly for powders with high surface area and low moisture content.

In addition, Cortez et al., highlighted the work of Robbin (2014) who developed a layered hard-panned coating method using natural blue anthocyanins as an alternative to synthetic blue dyes [30,76]. The process involves applying a sugar–calcium carbonate layer first, followed by layers containing anthocyanins and optional yellow pigments to create various colours, while preventing pH-induced colour changes. These coatings showed strong colour stability under moisture. Later, Robbin (2015) patented a similar method for brown coatings using purple carrot anthocyanins and turmeric yellow pigments, with pH adjustment critical for achieving brown hues [77]. The coatings matched synthetic browns closely in colour and demonstrated good stability. These innovations offer natural alternatives to synthetic hard-panned candy colourants [77].

### 4.4. Light-Blocking Packaging

Light, especially in the ultraviolet (UV) and visible spectrum, can induce pigment degradation through photochemical and photooxidative mechanisms. This is particularly critical for colourants such as curcumin, anthocyanins, and carotenoids, which are sensitive to light-induced isomerisation and oxidation. The mechanism of UV-light protection lies in the ability of barrier additives or fillers to absorb, reflect, or scatter incident UV radiation, thereby reducing the transmittance of harmful wavelengths (200–400 nm) through the packaging film. This mechanistic shielding prevents photochemical reactions such as isomerisation, oxidation, and pigment degradation, ultimately maintaining colour stability and nutrient integrity in foods [76,77].

Roy et al., highlighted that incorporating UV-absorbing additives or fillers into packaging materials can substantially reduce light transmission in the 200–400 nm range, thereby preserving the integrity of light-sensitive compounds [76]. In a practical application, Assis et al., investigated cellulose acetate films embedded with carotenoids (lycopene, norbixin, and zeaxanthin) and demonstrated that norbixin-containing films preserved up to 72% of vitamin B2 by acting as UV–Vis barriers. These films also exhibited pigment-specific release behaviour into food simulants, with lycopene showing the highest migration into lipid-rich environments [77]. Such active packaging solutions therefore provide dual functionality: protecting internal food constituents while simultaneously serving as delivery systems for bioactives.

### 4.5. Storage Condition Control

The effectiveness of physical stabilisation is ultimately influenced by post-processing storage conditions, particularly temperature, humidity, and oxygen levels. Oxygen accelerates anthocyanin degradation through oxidative cleavage of the phenolic rings, leading to pigment browning and colour loss, especially under heat [78]. While oxygen-restricted conditions help retain anthocyanins during cold storage, brief exposure to high oxygen levels may temporarily boost anthocyanin content, though this effect diminishes over time. Post-extraction stabilisation of natural colourants can be enhanced through strategies such as controlled atmospheres and drying techniques [78]. Temperature affects pigment stability both via thermal denaturation of sensitive bioactives and by enhancing enzymatic oxidation, whereas moisture promotes hydrolytic reactions that further degrade anthocyanins and carotenoids [78,79].

Ghidouche et al., (2013) proposed a novel accelerated shelf-life testing model for natural colourants using high-intensity light irradiation at controlled temperatures [79]. The mechanism relies on simulating photooxidative stress, where light energy induces excited states in pigment molecules, leading to formation of reactive oxygen species (ROS) that attack chromophores, resulting in colour fading [79]. By applying irradiation acceleration factors (QL), the study successfully established correlations between normal and accelerated light-induced degradation, enabling faster and predictive evaluation of pigment stability in aqueous systems.

Salazar-Orbea et al., (2023) further highlighted the importance of storage over processing when assessing polyphenol retention in fruit purees [80]. In strawberries, anthocyanin degradation occurs primarily via enzymatic oxidation (polyphenol oxidases) and non-enzymatic hydrolysis, both of which are accelerated by high temperatures and prolonged storage, whereas in apple puree, thermal processing induced partial polymerisation of flavonols, affecting colour stability [80]. The study used strawberry and apple purees to evaluate how industrial-scale processing methods such as freezing, high-pressure processing (HPP), and thermal treatments along with storage at −20 °C, 4 °C, and 24 °C for up to 12 months, affect bioactive phenolics and colour. Proanthocyanidins were the most stable phenolic group across both fruits, while anthocyanins showed the greatest degradation. Apple flavonols and dihydrochalcones remained relatively stable, whereas ellagitannins in strawberries declined significantly over time. Overall, the impact of storage versus processing varied by fruit type and phenolic class, with storage temperature and duration being especially critical for preserving polyphenols in delicate fruits like strawberries.

Howard et al., (2014) and Ranjbar Nedamani (2022) showed that strawberry purée processed and stored under nitrogen or CO_2_ retained more anthocyanins and colour stability over eight weeks compared to samples exposed to air [81,82]. This preservation occurs because inert gases displace oxygen, reducing oxidative reactions at the molecular level, and minimising ROS formation that would otherwise cleave pigment chromophores [82].

Reducing oxygen exposure is a key strategy in preserving food colour and quality. Techniques such as vacuum packaging, modified atmosphere packaging (e.g., using nitrogen or carbon dioxide), bottling under inert gases, and applying impermeable films or edible coatings help limit oxidation. Edible coatings and barrier films act mechanistically by physically restricting oxygen diffusion and moisture transfer, thereby slowing oxidative and hydrolytic degradation pathways of embedded pigments [44]. Increasing attention is also being given to packaging materials enhanced with antioxidants and antimicrobials such as chitosan, BHA, BHT, lysozyme, nisin, tocopherols, and natamycin to further improve shelf life. These additives scavenge ROS or chelate metal ions that catalyse oxidative reactions, providing an additional chemical stabilisation mechanism [44]. Table 8 presents additional studies on these physical techniques.

1. **Comparative Assessment of Techniques for Stabilising Natural Food Colourants**

While various chemical, physicochemical, and physical methods have been demonstrated to enhance pigment stability, it is important to compare these approaches in terms of their effectiveness, scalability, and economic feasibility to guide practical food industry applications. When comparing stabilisation strategies, there are notable differences in effectiveness, scalability, and cost. Co-pigmentation and metal ion complexation are highly effective for enhancing anthocyanin stability but may have limited scalability and moderate-to-high costs due to the need for purified co-pigments and precise formulation control [34,35,36,37,38,39]. Microencapsulation, including spray-drying and nanoencapsulation, offers high effectiveness and moderate scalability, with costs dependent on the choice of encapsulating material [49,50,51,52,53,54,55]. Hydrocolloid complexation is moderately effective but highly scalable and relatively low-cost, making it suitable for industrial applications [38,57,58]. Pickering emulsions provide excellent protection of pigments but require careful selection of stabilising particles, resulting in moderate scalability and higher production costs [61,62]. Overall, combining modern physicochemical and natural chemical methods can provide synergistic benefits, balancing stability, feasibility, and cost-efficiency.

2. **Advanced Food Processing Methods to Enhance Stability of Natural Food Colourants**

Recent advances in food processing technologies offer additional, innovative strategies to further improve pigment retention and functionality. The following section delves into advanced food processing techniques, outlining their mechanisms, advantages, and potential applications in enhancing the stability of natural food colourants across various food products. Additionally, Table 9 summarises the comparative evaluation of these stabilisation methods, highlighting key differences in their effectiveness, applicability, and limitations.

**High Pressure Processing**

High Pressure Processing (HPP) is a non-thermal technology that significantly impacts food pigments, particularly in enhancing colour retention [94]. The mechanism of HPP lies in its ability to inactivate spoilage microorganisms and degradative enzymes through high hydrostatic pressure (100–600 MPa) without applying heat, thereby preventing thermal denaturation and oxidation of pigments [93]. By preserving the native structure of bioactive molecules, HPP maintains both colour and nutritional quality, making it increasingly favoured over traditional thermal processing [92,94].

HPP has been shown to enhance the stability of anthocyanins, naturally occurring polyphenolic pigments in fruits and vegetables, by promoting molecular interactions with biopolymers such as proteins and polysaccharides. These interactions result in protective complexes where non-covalent bonds hydrogen bonding and hydrophobic interactions—stabilise the anthocyanin molecules against pH fluctuations, oxidation, and thermal stress [95]. The process can induce mild cross-linking and molecular reorganisation, enhancing pigment thermal tolerance and stability under alkaline conditions. However, applying excessively high pressures may disrupt these interactions, breaking hydrogen bonds and hydrophobic forces, leading to complex dissociation and increased susceptibility to degradation, including anthocyanin ring opening and formation of colourless derivatives [95].

HPP has also been shown to improve the stability of C-Phycocyanin (C-PC), a blue pigment, through pH-dependent mechanisms. At acidic pH (~3.0), the native conformation of C-PC polypeptide subunits is preserved under high pressure, minimising aggregation and colour loss. Conversely, extreme pressure can induce conformational changes, destabilising pigment structure and reducing colour integrity [96].

HPP demonstrates the importance of optimising processing parameters such as pressure magnitude, duration, and pH to maximise pigment stability while avoiding structural disruption. Table 10 shows additional studies on the effects of HPP on various pigments.

**Ultrasound**

Ultrasound processing has emerged as a significant non-thermal technique in the food industry, particularly for preserving food pigments and enhancing colour retention [97,98,99,100,101,102]. The mechanism of ultrasound lies in acoustic cavitation, where the formation and collapse of microbubbles generates localised high temperatures and pressures. These microenvironments enhance pigment extraction and can stabilise pigments by promoting interactions with surrounding matrices while avoiding bulk heating, which minimises thermal degradation of heat-sensitive compounds such as anthocyanins and carotenoids [102]. Ultrasound is commonly used in juice processing as a non-thermal alternative to pasteurisation, effectively reducing microbial load while maintaining quality [114].

Studies indicate that ultrasound can maintain or even improve the colour properties of food products. For example, Oner (2023) [112] reported that US treatment (120 µm, 24 kHz, up to 2 min at 20 °C) caused no significant colour changes in avocado dressing and green juice. The preservation mechanism is linked to low-temperature processing combined with cavitation-induced shear forces, which prevent pigment degradation while slightly increasing lightness in green juice, giving a brighter appearance [112]. Ultrasound effectiveness depends on optimising parameters such as frequency, power, treatment duration, and temperature [112,114].

Ultrasonic treatment of blueberries (anthocyanins) at 20 W/g for 20 min using a 45 kHz bath resulted in increased L*, a*, and b* values, reflecting enhanced colour intensity and brightness. In Cornelian cherry anthocyanin extraction, the Ohmic Heating-Assisted Ultrasound (OHAU) method (320 W, 35 kHz) produced the highest redness (+a*) and total phenolic content, while lightness (L*) decreased slightly. These improvements are mechanistically attributed to cavitation-induced cell wall disruption, which enhances pigment release, and the maintenance of pigment stability by reducing exposure to oxygen and thermal stress [101,102].

When used with gentle heat (typically below 50 °C), ultrasound provides a viable alternative for juice preservation without compromising sensory qualities such as taste, colour, and aroma [101]. The mechanism allows pigments to remain structurally intact while improving extractability and functional stability. Despite these advantages, standardising treatment conditions across food matrices remains challenging, and further studies are required to evaluate the long-term effects on pigment stability and food quality. Table 10 shows additional studies on ultrasound treatments.

**Pulse Electric Field**

Pulsed Electric Field (PEF) stabilises pigments by inducing electroporation in plant cell membranes, which facilitates pigment release while preserving molecular integrity. The high-voltage pulses create temporary pores in membranes, enhancing diffusion of anthocyanins, carotenoids, and other bioactive compounds into the surrounding medium without applying heat. This minimises pigment degradation caused by thermal stress and oxidation, while maintaining antioxidant activity and colour stability [88,89,115].

PEF is a modern, non-thermal processing method in which food placed between electrodes in a treatment chamber is exposed to brief, high-voltage electric pulses lasting from nanoseconds to milliseconds. The electroporation effect effectively inactivates spoilage microorganisms, offering microbial safety while maintaining nutritional and visual qualities [88]. By avoiding high temperatures, PEF ensures minimal loss of sensitive colour compounds, enhancing colour retention and improving the yield of natural pigments compared to conventional thermal extraction methods. For instance, in fruit juice processing, PEF treatment has been reported to retain up to 90% of vitamin C content, substantially higher than typical thermal processing [87,113,116].

While PEF shows promise for maintaining colour and nutritional quality, scalability and long-term effects on diverse food matrices remain areas requiring further research. Optimising parameters such as field strength, pulse duration, and treatment time is essential for consistent pigment stability and industrial application. Table 10 summarises additional studies investigating PEF treatments for food colourants.

**Irradiation Techniques**

Irradiation techniques stabilise food pigments primarily through the inactivation of degradative enzymes and modulation of molecular structures. Electron beam, gamma radiation, and cold plasma treatments reduce enzyme activity (e.g., chlorophyllase, Mg-dechelatase) and disrupt pathways that lead to pigment oxidation or hydrolysis. Additionally, these methods can induce mild structural changes in pigment-protein complexes, which may enhance pigment retention and colour stability while also improving microbial safety [85,113].

Irradiation methods, particularly electron beam and gamma radiation, have shown promise in preserving the stability of pigments such as chlorophylls, carotenoids, and anthocyanins during storage, thereby extending the shelf life of food products [85]. For example, in irradiated dried lavers, carotenoid content remained high after 12 weeks, while chlorophyll demonstrated better stability at a 7 kGy dose [84,85,86,87,88,89,90,91,92,93,94,95,96,97,98,99,100,101,102,114]. Anthocyanins in black rice flour were most stable at a 1 kGy dose, suggesting that lower irradiation levels can effectively preserve bioactive compounds during storage [85,117,118].

While irradiation can improve pigment stability, excessive doses may degrade certain pigments, emphasising the importance of optimising protocols to balance colour retention, bioactive preservation, and food safety. Table 10 summarises additional studies investigating irradiation techniques for food colourants.

**Cold Plasma**

Cold plasma (CP) preserves food pigments primarily through the generation of reactive species, including reactive oxygen species (ROS) and reactive nitrogen species (RNS), which interact with pigment molecules to stabilise them while inactivating degradative enzymes. These species—such as ozone (O_3_), singlet oxygen (^1^O_2_), superoxide anion (O_2_^−^), hydrogen peroxide (H_2_O_2_), nitric oxide (NO), and peroxynitrite (ONOO^−^) are formed when gases like air, oxygen, or nitrogen are ionised under electric fields. CP also disrupts cell membranes, enhancing pigment extraction into the extracellular space and improving colour retention [91,109,110,111,119,120].

CP has emerged as a promising non-thermal method to enhance the stability of food pigments, supporting both consumer acceptance and nutritional quality [93]. It preserves the optical properties of various food products while minimising degradation of sensitive pigments. Studies indicate that CP treatment generally results in minor pigment losses, with some cases showing improved preservation and extraction yields [121]. CP treatment also affects pigment stability. Bussmann et al., reported that barley leaves (*Hordeum vulgare* cv. Kosmos) treated with plasma-activated water (PTW) exhibited enhanced chlorophyll concentration, improved quantum yield, and elevated total ascorbate levels [90]. In kiwifruit juice, chlorophylls were more heat-sensitive than carotenoids, with slight reductions observed during CP treatment. Application of pin-to-plate CP at 180 V for 5 min enhanced carotenoid extraction yields, although the associated pH decrease could destabilise pigment-protein complexes, potentially leading to carotenoid degradation over time [122,123].

While CP can induce minor colour changes, it often maintains overall colour quality, with effectiveness dependent on food type and treatment parameters, requiring optimisation on a case-by-case basis [122,123]. CP is particularly advantageous for preserving minimally processed foods, aligning with consumer demand for fresh and nutritious options [122]. Despite its benefits, further research is needed to fully understand its effects on food pigments and optimise applications in food processing. Table 10 shows additional studies on the application of CP treatment on food colourants.

3. **Comparison of Advanced Food Processing Methods for Enhancing the Stability of Natural Food Colourants**

HPP is highly effective in retaining pigments and nutritional compounds, but its high equipment and operational costs limit scalability to niche, high-value products [94]. US offers moderate cost and good pigment retention, yet its lower throughput makes it less practical for large-scale operations [77,78,79,80,81,82,83,84,85,86,87,88,89,90,91,92,93,94,95,96,97,98,99,100,101]. PEF provides rapid, non-thermal treatment with minimal pigment degradation, but the high capital investment and complex equipment requirements remain barriers for industrial use [88,89,90]. Irradiation techniques are scalable and cost-efficient, yet consumer acceptance and regulatory restrictions hinder widespread adoption despite proven stability benefits [85,86,87,88,89,90,91,92]. CP is emerging as a low-cost, environmentally friendly option with promising results in pigment stabilisation, although scalability and long-term colour stability need further validation [109,120]. While these methods enhance pigment stability, their effectiveness, scalability, and cost vary considerably, requiring context-specific selection.

4. **Regulatory Uncertainty of Advanced Stabilisation Technologies**

Advanced stabilisation methods, including cold plasma, high-pressure processing, and nano- or microencapsulation, offer significant potential to enhance the stability, colour retention, and bioactivity of natural food pigments. However, the regulatory environment for such non-thermal and emerging technologies remains fragmented and evolving. Cold plasma, for instance, is recognised for its non-thermal, energy-efficient ability to inactivate microbes and preserve pigment integrity, yet regulatory frameworks for its use in food processing differ across regions. Authorities often require extensive safety evaluations, demonstration of consistent product quality, and validation of residual effects before granting approval for commercial applications. This uncertainty can delay industrial adoption despite proven laboratory-scale efficacy [5,53,78].

5. **Industrial Implications of Stabilised Natural Pigments**

Translating stabilisation strategies from the laboratory to industrial settings requires more than technical feasibility; it involves assessing economic viability, process integration, and operational efficiency. While advanced techniques such as micro- and nanoencapsulation, co-pigmentation, and non-thermal treatments have demonstrated efficacy in preserving pigment stability, their industrial adoption depends on the availability of scalable equipment, control over process parameters, and minimisation of energy consumption [29,30].

In practice, pilot-scale evaluation is crucial to determine optimal operational conditions, such as encapsulation carrier composition, pressure levels, or treatment duration, to ensure consistent pigment performance and sensory quality across large production batches. Additionally, industrial implementation can benefit from utilising agro-industrial by-products as pigment sources, supporting circular economy principles while reducing costs and environmental impact [29].

Finally, strategic alignment with regulatory frameworks and consumer expectations is essential for commercial success. Companies must consider safety standards, labelling requirements, and public perception of novel processing technologies to facilitate market acceptance. By addressing these industrial, economic, and regulatory aspects, stabilised natural pigments can move from laboratory innovation to practical, large-scale applications, enabling the production of visually appealing, high-quality, and sustainable food products [30,31].

6. **Sensory Impact and Consumer Acceptance of Stabilised Natural Colourants**

Consumer acceptance is critical for the commercial success of natural food colourants, particularly when advanced stabilisation methods such as nano- and microencapsulation, HPP, and CP are used. These techniques enhance pigment stability while preserving key sensory attributes, including appearance, flavour, and overall acceptability. Encapsulation reduces off-flavours associated with concentrated plant extracts, improving sensory scores in beverages and dairy products [123,124]. HPP maintains anthocyanin colour intensity and fresh-like aroma in fruit juices, leading to higher panellist ratings compared to thermally treated products [125,126]. Cold plasma has been shown to retain the vividness of betalains and carotenoids without introducing undesirable flavours, supporting positive consumer perceptions [127].

Despite these advantages, novel processing approaches may trigger skepticism due to perceived unnaturalness or safety concerns. Transparent communication regarding the natural origin of pigments, non-thermal processing, and additional benefits such as enhanced nutritional quality, sustainability, and shelf-life extension is essential to build consumer trust. Evidence shows that products with stable, vibrant colours and unaltered flavour or aroma consistently achieve higher liking and purchase intent. Therefore, integrating advanced stabilisation strategies is vital not only for extending shelf life but also for improving sensory appeal and overall marketability of naturally coloured foods [124,125].

7. **Environmental and sustainability considerations**

Environmental and sustainability considerations are becoming increasingly central in the application of natural food colourants. The valorisation of by-products from fruit, vegetable, and plant processing for pigment extraction represents a practical example of circular economy principles, reducing food waste while enhancing resource efficiency. In parallel, stabilisation techniques such as microencapsulation and non-thermal processing not only improve pigment stability but also reduce the energy demands commonly associated with conventional thermal methods, thereby contributing to more sustainable production practices. Incorporating waste streams into pigment production, coupled with the adoption of environmentally friendly processing strategies, directly supports industry objectives for sustainable food systems and strengthens commitments to corporate social responsibility [125,126].

Beyond environmental benefits, stabilisation methods exert a direct influence on the sensory qualities of food products enriched with natural pigments. Attributes such as hue, colour intensity, and visual uniformity are key drivers of consumer perception and acceptance. Encapsulation strategies including micro- and nanoencapsulation are particularly effective in protecting sensitive pigments against degradation while simultaneously masking undesirable flavours or bitterness that can be associated with concentrated natural extracts. Similarly, non-thermal technologies such as high-pressure processing and cold plasma preserve the integrity of colour compounds without imparting heat-related off-flavours or aroma losses, thereby offering an advantage over traditional thermal treatments. Sensory evaluations consistently demonstrate that consumers show a preference for products with stable, vivid, and naturally derived colours, highlighting the need to optimise both functional stability and sensory attributes in the development of colourant-enriched foods [28,127,128].

8. **Knowledge Gaps**

Despite rapid advances in the stabilisation of natural food colourants, several critical knowledge gaps remain that limit their broader application in the food industry. Much of the current research has focused on simplified model systems, yet the stability and functionality of pigments in complex food matrices remain insufficiently understood. Interactions with proteins, polysaccharides, lipids, minerals, and bioactive compounds can alter pigment structure, colour expression, and bioactivity, but systematic studies across diverse product categories are lacking. Similarly, while many investigations examine the effects of individual thermal and non-thermal processing methods (e.g., pasteurisation, HPP, PEF, CP), there is limited evidence on their combined or sequential application during industrial-scale food manufacturing and long-term storage [124,125,127].

Multiple stabilisation strategies including co-pigmentation, nano- and microencapsulation, protein–polysaccharide complexation, emulsification, antioxidant incorporation, and active packaging systems have shown potential for improving colour retention and functionality. However, the mechanistic understanding of these approaches is incomplete. For instance, the optimal combinations of pigments, co-pigments, and encapsulating or wall materials for different food categories are not yet established. Interactions within multi-pigment systems or in the presence of reactive ingredients (e.g., reducing sugars, ascorbic acid, transition metals) remain poorly characterised. Moreover, while encapsulation and co-pigmentation improve pigment stability, their influence on bioavailability, digestibility, and health-promoting functions is not fully elucidated, as most studies are based on in vitro conditions rather than in vivo or clinical investigations.

Other important gaps relate to regulatory, consumer, and sustainability aspects. The acceptance of advanced stabilisation techniques particularly nanoencapsulation and non-thermal technologies may be constrained by differing regional regulations and consumer concerns around safety and naturalness. From a sustainability perspective, strategies for pigment recovery from agri-food by-products and the development of energy-efficient stabilisation technologies are promising, but robust life-cycle analyses and techno-economic assessments are scarce. Finally, integrated or multi-hurdle approaches such as combining encapsulation with non-thermal processing or antioxidant supplementation remain underexplored. Addressing these gaps through interdisciplinary research that links stability, sensory quality, health outcomes, and environmental performance is essential to unlock the full potential of natural colourants in the food sector.

## 5. Conclusions and Future Perspective

The shift towards natural food colourants reflects growing consumer demand for healthier, safer, and more sustainable food products. Despite the benefits, natural pigments suffer from instability and susceptibility to environmental factors which limit their widespread industrial application. This review highlights various novel techniques that improve the stability, bioavailability, and colour retention of natural pigments, underscoring their potential as viable alternatives to synthetic dyes.

Recent advancements in technologies, including nanoencapsulation, co-pigmentation, and the use of hydrocolloids, alongside non-thermal processing techniques such as high-pressure processing, ultrasound, irradiation, and cold plasma, have demonstrated significant potential in enhancing the stability and functionality of natural pigments. Collectively, these technologies provide effective means to stabilise and preserve colour quality and nutritional value of pigments in processed foods while maintaining clean-label attributes.

Moving forward, further research and investment in these technologies will be essential to overcoming existing challenges, promoting the large-scale adoption of natural pigments, and meeting the evolving demands. Future studies should aim to optimise processing parameters to maximise pigment retention while minimising energy consumption and production costs. Combining these technologies with other stabilisation methods, such as co-pigmentation and hydrocolloid incorporation, may lead to synergistic effects that further improve pigment stability. By addressing these areas, the food industry can unlock the full potential of natural pigments, paving the way for healthier, more sustainable, and visually appealing food products that meet evolving consumer preferences.

## Figures and Tables

**Table 1:** Comparison between different types of food colourants [2,3,12].

Criteria	Natural	Inorganic (Mineral-Based)	Synthetic	Nature-Identical	Microbial
Source	Extracted directly from natural sources (plants, animals, insects, microbes)	Derived from minerals (e.g., titanium dioxide, iron oxides)	Fully chemically synthesised, not found in nature	Chemically synthesised to be structurally identical to natural compounds (e.g., β-carotene)	Produced through microbial fermentation (e.g., Monascus pigments)
Solubility & Stability	Less stable; affected by heat, light, pH, and oxidation	Very stable, insoluble in water, used for opacity	Highly stable under various processing conditions	More stable than natural colourants, depends on the specific compound replicated	Moderately stable, can degrade under certain processing conditions
Colour Range	Limited and often variable	Limited to whites and earth tones	Wide range with intense and uniform colours	Moderate range, depends on the specific compound replicated	Moderate, includes reds, yellows, and some blues and violets
Nutritional/Health Value	May offer antioxidants or other bioactive compounds	No nutritional value, purely functional	No inherent nutritional benefits; some associated with adverse health effects	May retain some beneficial properties (e.g., vitamin A activity of β-carotene)	Potential health benefits, some show antioxidant or anti-inflammatory properties
Safety & Regulation	Considered safer and ‘clean-label’; requires validation	Approved in limited use, some concerns over nanoparticle forms	Approved with strict limits, some are banned or restricted due to health concerns	Approved by regulatory bodies, often perceived as safer than synthetic	Generally considered safe, still under regulatory evaluation
Consumer Perception	Highly favoured by clean-label and health-conscious consumers	Neutral to negative, seen as artificial or industrial	Increasingly rejected by health-conscious consumers	Moderately accepted, perception varies	Gaining interest due to sustainability and perceived natural origin
Cost	Expensive due to sourcing and extraction	Low to moderate, cost-effective but limited in colour range	Low cost and economical for large-scale production	Moderate; cheaper than natural, more expensive than synthetic	Variable, cost decreasing with fermentation advances
Application Suitability	Limited due to sensitivity to processing conditions	Used mainly for opacity or earth tones (e.g., candies, coatings)	Highly versatile, used across a broad range of products	Suitable for more products than natural but not as universal as synthetic	Promising for beverages, dairy, and functional foods
Environmental Impact	Generally sustainable and biodegradable if ethically sourced	Mining impacts possible, concerns over nanoparticles	Less sustainable, may involve petrochemical derivatives	More controlled synthesis, lower impact than synthetic	Renewable and environmentally friendly via biotechnology
Allergenicity	Some (e.g., carmine) may trigger allergies	Low allergenic potential, generally inert	Some cause allergic reactions (e.g., tartrazine)	Low risk when purified, usually hypoallergenic	Low allergenicity, generally well tolerated

**Table 2:** Classification of natural pigments based on solubility, source, and application in food [1,2,3,4].

Colourant	Solubility	Colour Range	Sources	Applications
Anthocyanins	Water-soluble	Red, blue, purple, yellow	Fruits, vegetables	Beverages, jams, dairy, confectionery
Carotenoids	Fat-soluble	Yellow to purple	Algae, plants, animals, bacteria, fungi	Food colourants, used in pickles, sauces
Betalains	Water-soluble	Red, yellow	Plants in Caryophyllales (e.g., beetroot, prickly pear)	Dairy (e.g., yoghurt), beverages
Chlorophylls	Fat-soluble	Green	Algae, cyanobacteria, plants	Health supplements, detox beverages
Phycocyanin	Water-soluble	Blue	Cyanobacteria (e.g., Spirulina), red algae	Cosmetics, foods, nutraceuticals, biotechnology
Astaxanthin	Water-insoluble (mainly protein-bound pigments)	Red, pink, brown	Haemoglobin and myoglobin from crustacean shells	Used in processed meats (sausages, ham), seafood colour enhancement, cosmetics (e.g., carmine), and limited dairy applications
Carmine	Water and alcohol soluble	Deep red to crimson	Cochineal insects (producing carminic acid, i.e., carmine)	Beverages, yoghurts, candies, bakery, cosmetics
Kermesic acid	Water and alcohol soluble	Deep red to crimson	Derived from Kermes	Cosmetics, Sausages, meat product, meat products, seafood, confectionery, beverages, seasonings
Lac dye	Water and alcohol soluble	Crimson to burgundy reds to deep purples	Derived from insect Laccifer lace/kerria	Sausages, meat product, meat products, seafood, confectionery, beverages, seasonings
Tyrian purple	Water and alcohol soluble	Reddish-purple	Derived from Molluscs	Sausages, meat product, meat products, seafood, confectionery, beverages, seasonings

**Table 3:** Comparative Overview of Selected Food Colourants with Regulatory Limits and Codes.

Colourant	Max Allowed Concentration	Regulatory Authority	Code (F/FD&C/INS)	Regio/Reference
Allura Red	200 mg/kg	FDA	FD&C Red No. 40	USA [13,21]
Betalains	No specific limit	EFSA	E162	EU [7,14]
Chlorophyll	10%	FSSAI	E140	India [15,16,17]
Curcumin	95%	FSANZ	INS 100	Australia/NZ [22]
Plant-based Carotenes	5%	EFSA	E160a	EU [7,14]

**Table 4:** Advantages and disadvantages of natural food colourants.

Colourant	Source	Advantages	Disadvantages	References
Anthocyanins	Fruits, vegetables (e.g., berries, red cabbage)	- Water-soluble - pH-dependent colour variation - Antioxidant, anti-inflammatory, anti-obesity, antidiabetic, anticancer	- Unstable to pH, temperature, and light - Colour shift issues - Can affect flavour at high doses	[4,24]
Carotenoids	Carrots, tomatoes, paprika, algae	- Fat-soluble - Stable in acidic environments - Health-promoting antioxidant role	- Water-insoluble - Sensitive to light and oxidation - Requires emulsification	[22]
Betalains	Beetroot, prickly pear	- Water-soluble - Stable in pH 3–7 - Strong antioxidant and anti-nitrative stress benefits	- Light and heat sensitive - Flavour changes at high levels - Possible consumer rejection	[25,26]
Chlorophylls	Green leafy vegetables, algae	- Natural green hue - Detoxifying and gut-supporting	- pH- and heat-sensitive - Mg replacement leads to colour loss (pheophytins)	[4]
Phycocyanin	Cyanobacteria, red algae	- Bright blue - Immune-boosting and anti-cancer - Cosmetic and nutraceutical applications	- Light, pH, and heat sensitive - Cost-intensive extraction	[2]
Curcumin	Rhizome of *Curcuma longa*	- Yellow pigment - Antibacterial, antioxidant, anti-inflammatory - Preservative properties	- Light-sensitive - Strong flavour - Poor water solubility	[27]
Astaxanthin	Algae, yeast, crustaceans	- Powerful antioxidant - Reddish-orange - Used in aquaculture and health products	- Expensive natural extraction - Poor water solubility	[2]
Carmine	Insects (*Dactylopius coccus*)	- Bright red - Highly stable to heat/light/pH - Widely approved (E120)	- Not vegan/vegetarian - Allergenic potential - Cultural/religious concerns (e.g., kosher/halal)	[2]
Haem-Derived Colourants	Animal muscle tissue, blood	- Contributes red/pink colour to meat - Essential for colour in processed meats - Heatstable in some forms (nitrosylmyoglobin)	- Unsuitable for plant-based foods. - Regulatory and ethical concerns - Limited use outside meat products	[2]
Kermesic acid	Derived from Kermes	- No direct health benefits; generally safe but may cause allergic reactions; not suitable for vegetarians/vegans/kosher/halal diets	- Not vegan/vegetarian - Allergenic potential - Cultural/religious concerns (e.g., kosher/halal)	[2]
Lac dye	Derived from insect Laccifer lace/kerria	- No direct health benefits; generally safe but may cause allergic reactions; not suitable for vegetarians/vegans/kosher/halal diets	- Not vegan/vegetarian - Allergenic potential - Cultural/religious concerns (e.g., kosher/halal)	[2]
Tyrian purple	Derived from Molluscs	- No direct health benefits; generally safe but may cause allergic reactions; not suitable for vegetarians/vegans/kosher/halal diets	- Not vegan/vegetarian - Allergenic potential - Cultural/religious concerns (e.g., kosher/halal)	[2]

**Table 5:** Advanced characterisation techniques for natural food colourants: evaluation of stability and effectiveness.

Technique	Purpose/Parameter Evaluated	Examples/Application	Reference
UV–Vis Spectroscopy	Determines absorbance spectra, colour intensity, degradation kinetics under light/heat	Monitoring stability of anthocyanins and carotenoids under thermal and pH stress	[28]
HPLC–PDA/HPLC–MS	Separates and quantifies pigment components; detects degradation products	Analysis of carotenoids, betalains, anthocyanins in beverages and encapsulated systems	[29]
Fourier Transform Infrared Spectroscopy (FTIR)	Identifies functional groups, pigment–encapsulant interactions	Examining binding between natural pigments and biopolymers in encapsulation systems	[30]
Scanning Electron Microscopy (SEM)/Transmission Electron Microscopy (TEM)	Observes morphology, encapsulation structure, surface properties	Visualising micro/nano-capsule structure for carotenoids and anthocyanins	[31,32]
Thermogravimetric Analysis (TGA) & Differential Scanning Calorimetry (DSC)	Assesses thermal stability, degradation temperature, phase transitions	Stability evaluation of natural pigments in nano/microencapsulation systems	[30]
X-ray Diffraction (XRD)	Determines crystalline vs. amorphous structure of encapsulated systems	Characterising crystalline nature of pigment-loaded nanoparticles	[29]
Chromatographic fingerprinting & HPLC-based quantitative assays	Verification of pigment authenticity and content	Quantification of anthocyanins and carotenoids in model food systems	[31,32,33]

**Table 7:** Physicochemical stabilisation techniques for natural food colourants.

Technique	Primary Pigments Targeted	Mechanism	Applicability to Other Pigments	Condition Used	Reference
Microencapsulation	Carotenoids, Anthocyanins, Betalains, Chlorophylls	Physical entrapment of pigments within a protective coating material to shield from oxygen, light, and moisture.	Highly versatile; applicable to nearly all natural pigments.	Various drying techniques (spray, freeze); stabilised pigments under thermal, oxidative, and light exposure.	[63,64]
Nanoencapsulation	Anthocyanins, Carotenoids	Encapsulation within nanocarriers (e.g., ferritin, liposomes, caseins, chitosan) enhancing stability, controlled release, and bioavailability.	Applicable to a wide range of hydrophilic and hydrophobic pigments	Nanoparticles formed under controlled pH, surfactant ratios, or polymer concentrations; stable under high temp and acidic pH.	[54,65]
Nanoemulsions	Carotenoids, Hydrophobic pigments	Formation of stable nano-sized emulsions that protect pigments from oxidation and improve bioavailability.	Mainly hydrophobic pigments but adaptable for mixed pigment systems.	Particle size ~100–200 nm; storage stability for 30 days; high bioactivity retention.	[64,65]
Hydrocolloid Complexation	Betalains, Anthocyanins, Curcumin	Entrapment of pigments within a hydrocolloid matrix (e.g., pectin, alginate) providing physical barrier and solubility enhancement.	Applicable to water-soluble pigments sensitive to environmental stress.	Complexation with pectin, chitosan, and alginate; improved pigment colour and oxidative stability under ambient and thermal stress	[54,66]
Pickering Emulsions	Various natural pigments	Stabilisation of emulsions using solid particles forming a robust interfacial layer protecting pigments.	Broadly applicable, especially in emulsified food systems.	Stable emulsions formed with food-grade particles; enhanced antioxidant retention and release control.	[61]

**Table 8:** Physical methods for enhancing the stability of natural food colourants.

Physical Technique	Description	Examples/Applications	References
**Particle Size Reduction**	Milling or grinding pigments to smaller sizes to improve dispersion and uniformity.	Spray-dried carrot powders with compact structure retained 76–77% carotenoids, while porous freeze-dried forms showed up to 93% degradation.	[67]
**Spray Drying/Freeze Drying**	Physical removal of moisture from pigment extracts to reduce water activity and degradation.	Chlorophyll microencapsulated by SD and FD showed better thermal stability in freeze-dried form Pea water pigments preserved via SD/FD; Cistus creticus phenolics retained using inulin/maltodextrin.	[26,70,71,72]
**Edible Films and Coatings**	Applying physical barrier layers to protect pigments from oxygen, light, and moisture.	Colour degradation in red pepper correlated with packaging film permeability and moisture levels. Vacuum packaging most effective.	[57]
**Light-Blocking Packaging**	Packaging that physically blocks UV and visible light to prevent photo-degradation.	Norbixin-cellulose acetate films retained 72% of vitamin B2; carotenoid-embedded films slowed degradation under UV.	[76,77]
**Storage Condition Control**	Controlling temperature and humidity to physically slow down pigment degradation reactions.	Light-accelerated testing predicted shelf life of anthocyanins polyphenol retention in fruit purees was storage-dependent.	[79,80]

**Table 9:** Effects of novel processing methods on the stability of selected natural food colourants.

Processing Method	Natural Colourant	Effect on Stability/Properties	Reference
Ultrasound-Assisted Extraction	Anthocyanins (various fruits, e.g., berries, purple maize)	Improved extraction yield; enhanced stability by reducing processing time; potential preservation of antioxidant activity.	[83,84]
Irradiation (Gamma Irradiation)	Beetroot extract (Betalains)	Gamma irradiation at 5.0 kGy preserved pigment structure; effective microbial control without major colour loss.	[85,86]
Pulsed Electric Fields	Chlorophylls (Spinach pigments)	PEF improved extraction efficiency; slight degradation of chlorophyll depending on intensity and duration.	[87,88]
Novel Encapsulation (Micro/nanocapsules)	Curcumin, Anthocyanins	Improved thermal and light stability; enhanced solubility and bioavailability in food matrices.	[89,90]
Cold Plasma Processing	Anthocyanins (Blueberry, grape extracts)	Retention of colour with minimal degradation; non-thermal method suitable for sensitive pigments.	[91]
High-Pressure Homogenisation	Chlorophylls, Carotenoids	Improved dispersion in food systems; partial degradation possible at extreme conditions.	[92,93]

**Table 10:** Effects of ultrasonic technique, high pressure processing, cold plasma, pulse electric field and irradiation on the natural food colourants.

Advanced Technique	Mechanism	Observation	Reference
Pulse electric field	Pulsed electric fields (PEFs)-electric field strengths (0.4, 1.2 and 2 kV cm^−1^) and number of pulses (5, 18 and 30 pulses)	- Greatest enhancement occurred under 2 kV/cm and 30-pulse treatment. - Lycopene: Bioaccessibility increased by 132%, δ-Carotene: Increased by 2%, β-Carotene: Increased by 53%, γ-Carotene: Increased by 527%, Lutein: Increased by 125%	[97]
Irradiation	Gamma radiation at doses of 0, 1, 2, and 3 kGy.	- Gamma radiation improves stability of anthocyanins and phenolic compounds. - Storage leads to gradual losses in bioactive compounds.	[98]
Cold plasma	Ten minutes of treatment was performed at a discharge voltage of 80 kV and 80 kV RMS (root mean square), Time: 5 and 10 min,	- CAP did not affect cholesterol or lipid content in meat. - Higher peroxide and thiobarbituric acid reactive substances (TBARS) values were found for the treated samples indicated accelerated primary and secondary lipid oxidation in meat. - No changes in colour	[99]
Ultrasonic	Sweeping-frequency ultrasonic preprocessing (SFUP). Centre Frequency: 60 kHz, Sweeping Amplitude: ±1 kHz, Sweeping Period: 100 ms, Power: 25 W L^−1^	- SFUP increased OPI removal rate to 49.32%. - OPI through 80 mesh showed brighter colour and stability. - Sufficient OPI in M80 maintained stability with additives. - Greenness increases by 15.51%, Greater stability with SFUP in starch solution (0–0.6).	[100,101,102]
Ultrasonic	Ultrasound (200–500W), (15–90 min)	- Cy-3-glu stability decreased under power ultrasound treatment. - Longest half-life observed in 20% ethanol solution.	[103,104,105,106]
Ultrasonic	Ultrasonic treatment at 20, 28, and 40 kHz, power at 100 W for 20 min	- Strong evidence of brilliant colour with attractiveness in line with the b* value (28 and 40 kHz) - Higher monomeric anthocyanin concentration 40 kHz - Increased antioxidative ability due to surviving anthocyanins.	[107]
Ultrasonic	Ultrasound-150 W and 40 kHz for 5 s, 10 s, 30 s, 1 min, or 5 min	- Delphinidin retained at 47.44% in 1 min, Petunidin retained at 26.18% in 1 min. - Delphinidin is more stable than petunidin. - Ultrasound caused less degradation than microwave treatment.	[108]
Ultrasonic	20–40 Khz	- Ultrasonic technology improves juice quality and bioactive content. - Effective in inactivating microorganisms and enzymes for preservation.	[109]
Ultrasonic	Ultrasound-assisted bleaching (UAB). 25 kHz and with a working power of 1000 W, 40 and 80% amplitude	- Higher amplitude and temperature increased pigment removal. - Optimum condition for removal: Clay concentration: 1%, Temperature: 55 °C - Adsorption follows pseudo-second-order kinetics under ultrasound conditions.	[110]
Ultrasonic	Ultrasound irradiation—20 kHz, time—14 min and 28 min, Power—950 W.	- Ultrasound enhances co-pigmentation of caffeic acid in wine. - Improved wine colour and stability during storage.	[111]
High pressure processing	High-Pressure Processing (HPP)	- C-PC stability decreases at lower pH during HPP. - pH adjustment post-HPP reduces colour loss and aggregation.	[91]
High pressure processing	Effects of high pressure techniques on anthocyanin stability and comparing different pressure methods with traditional high temperature preservation	- High pressure techniques improve anthocyanin stability. - Traditional thermal methods cause significant pigment losses.	[66]
High pressure processing	HPP treatment of the phycocyanin-whey protein and carrageenan, 450 MPa and 600 MPa, pH-3,4 and 7	- HPP likely encapsulates the blue chromophore within the protein matrix, reducing oxidative damage. - Improved beverage quality and shelf life observed. - Stable at pH 3.0 during HPP with minimal colour loss and structural changes	[64,112]
Pulse electric field	Pulse electric fields (PEF) Batch system-5 pulses of 3.5 kV cm^−1^ (0.61 kJ kg^−1^) and were stored at 4 °C for 24 h.	- Showed an increase: isoferulic acid (97.7%), ferulic acid (29.2%) and feruloylquinic acid derivative (18.1%). - PEF caused structural changes enhancing release of antioxidants during digestion.	[65,113]

## Data Availability

No new data were created or analyzed in this study. Data sharing is not applicable to this article.
